# A Kernel-Based Approach for Biomedical Named Entity Recognition

**DOI:** 10.1155/2013/950796

**Published:** 2013-12-29

**Authors:** Rakesh Patra, Sujan Kumar Saha

**Affiliations:** Department of Computer Science & Engineering, Birla Institute of Technology, Mesra, Ranchi 835215, India

## Abstract

Support vector machine (SVM) is one of the popular machine learning techniques used in various text processing tasks including named entity recognition (NER). The performance of the SVM classifier largely depends on the appropriateness of the kernel function. In the last few years a number of task-specific kernel functions have been proposed and used in various text processing tasks, for example, string kernel, graph kernel, tree kernel and so on. So far very few efforts have been devoted to the development of NER task specific kernel. In the literature we found that the tree kernel has been used in NER task only for entity boundary detection or reannotation. The conventional tree kernel is unable to execute the complete NER task on its own. In this paper we have proposed a kernel function, motivated by the tree kernel, which is able to perform the complete NER task. To examine the effectiveness of the proposed kernel, we have applied the kernel function on the openly available JNLPBA 2004 data. Our kernel executes the complete NER task and achieves reasonable accuracy.

## 1. Introduction

Named entity recognition (NER) is a task of identifying the named entities (NE) from texts. In a text NEs are the pivotal elements to further contextualise the data. That is why NER has huge application in various text mining tasks.

In the last decade substantial amount of work has been done for development of NER systems in various languages and domains. Various systems have been developed for identifying NEs in biomedical domain [1–5]. Most of these systems are based on machine learning algorithms. In these machine learning based approaches, the problem is viewed as a classification problem and a classifier is trained using annotated data and a set of features. The efficiency of these systems mostly depends on effectiveness of the assembled feature set.

Support vector machines (SVM) is one of the most efficient and popular machine learning approaches which are being used in NER and other related tasks. It is basically a binary classifier which finds separating hyperplane with a large margin. That is why it is also known as a large margin classifier. Here the data (in NER task-words) is represented in the form of feature vectors. The similarity between two words is computed as the dot product between the corresponding vectors. The features used are mostly binary, which takes value one when that feature satisfies for the current word and zero otherwise. In the NER task most commonly used features are the current and surrounding words, suffix and prefix information of the words, and so on. A previous word feature can take any value from the lexicon (all unique words in the corpus) which designates the previous word. In this way only to the feature “previous word”, a range of binary features in feature space is included (total number of unique words). Similarly binary representation is there for other features also. This way, the total number of features for the NER task becomes huge. So the computation of dot product between the vectors is a tedious task.

Alternatively, the similarity between the words can be measured by using an appropriate kernel function. A kernel function helps in establishing an implicit relation between two words by mapping them into an alternative dimensional feature space. This saves time and effort of explicitly selecting features into the feature set for improving the overall efficiency. In the literature we found that various kernel functions have been proposed and used in various text processing tasks. Most of these techniques compute the distance between two sequences by finding the number of similar subsequences. In *string kernel* the distance between two words is computed by measuring the similarity in character sequences [6]. The *tree kernel* finds the distance between two trees by finding the similarity between their tree fragments [7]. Several variations of tree kernel are there, among which *subtree kernel*, *subset tree kernel*, and *partial tree kernel* are popular [8].

In the literature we observe that the use of task-specific kernel for NER task is limited. String kernel finds similarity between two words by computing character similarity and therefore not much effective in NER task. Tree kernel considers a sentence as an entity and finds a tree representation (e.g., parse tree) of the sentence in order to find the similarity. But in NER task the distance between the individual words has to be computed. Therefore the conventional tree kernel is not directly applicable in this task. We observe that in a few NER systems the tree kernel has been applied for boundary detection or reannotation. There the task is viewed as a two-phase process; the first phase identifies the class of the words and the second phase detects the boundary. In those tasks, for entity identification other machine learning classifiers like CRF are used and the tree kernel is used for named entity boundary detection [9] or reannotation [10].

In this paper we have proposed a novel kernel function for the NER task which is tangled to do both named entity identification and named entity boundary detection. This kernel will execute the complete NER task on its own. The design of the kernel is motivated by the tree kernel.

Similar to the tree kernel, the proposed kernel also uses a tree representation to the sentences. But instead of considering the whole tree we focus on the subtrees where the target word and its surrounding words are present. Here the target word is represented by a tree fragment where only the target and its surrounding words are present. To these fragments we also include additional marker nodes as parents of the target words. We name the proposed kernel as the “sliding tree kernel.”

The proposed kernel is tested for biomedical named entity recognition task using the popular JNLPBA 2004 data [11]. Experimental results and related comparisons demonstrate the efficiency of the sliding tree kernel in the NER task. The background, details of the kernel, and experiment results are presented in the subsequent sections of the paper.

## 2. Early Works on Biomedical NER

NER task is the primary task in biomedical domain. Many systems using various supervised learning classifiers, like hidden Markov model [1, 12], maximum entropy [3, 13], conditional random Field [14], SVM [4, 15], and so forth, have been proposed. A supervised learning classifier for NER task learns its classification model using a training data. In biomedical domain a few annotated NER data are openly available, for example, JNLPBA 2004 data.

Several systems participated in JNLPBA 2004 shared task. Among these, the highest accuracy was achieved by the system developed by Zhou and Su which produced an *F*-score of 72.55 [2]. This system used HMM and SVM with some deep knowledge resources. Without the domain knowledge the reported *F*-score of the system was 60.3. The addition of domain POS information increased the *F*-score to 64.1. Deep domain knowledge like name alias resolution, cascaded NE resolution, abbreviation detection, and external name dictionaries, when integrated in the system, raised the *F*-score to 72.55. Song et al. used SVM in their development. They expanded the corpus using a set of virtual examples which require some domain knowledge on the training data. They achieved a final *F*-score of 66.28 using CRF, SVM, postprocessing, and virtual samples. The baseline system achieved an *F*-score of 63.85 using SVM [15].

Among all the classifiers it has been found in the literature that SVM is more efficient as a classifier. The ease and efficiency in implementation of kernel techniques into the classifier is the most attractive part of SVM [16]. NER task has much been explored for many decades. There have also been language independent NER systems which use SVM as classifier [17].

Seeing the advantage of kernel techniques over extensive feature based model, lots of kernel techniques have been developed over the years. String kernel [6], tree kernel [7], clustering based kernel [18], kernel-based reranking [10], dependency tree kernel [19], tagging kernel [9], and so on are very few among all the kernel methods proposed for various text analysis tasks. Unfortunately all the kernels proposed for various text processing tasks are not applicable in the NER task.

Vanschoenwinkel [17] proposed a *polynomial overlap kernel* for language independent NER. This system showed an average *F*-score of 71.00%. For NER, tree kernel has never been directly used for named entity word identification. Nguyen et al. [10] used tree kernel to reannotate the examples using kernel-based reranking. At first conditional random field model with polynomial kernel was used to generate list of top *n* annotation candidates and this list was reranked with additional features using tree kernel. Collins and Duffy [9] used tree kernel for named entity boundary detection and left identification of NE to a separate stage of processing.

Looking into the various literatures, tree kernel has never been used to do complete NER task. It has always been partially used for NER subtasks like named entity boundary detection or reannotation.

## 3. Methods

### 3.1. Support Vector Machine

SVM is a supervised learning classifier. The cost estimation function in SVM gives special emphasis on avoiding overfitting by looking for global optima. This leads to an optimum hyperplane. That is why it is also known to be a large margin classifier. By default SVM is a binary classifier. But it can be utilized for multiclass classification by using one-vs-all method.

Of any domain, $\vec{X}{}_{i}$ to a classifier, the empirical data is represented in

(1) $$\begin{array}{c} \left(x_{1},y_{1}\right),\ldots ,\left(x_{m},y_{m}\right)\in \vec{X}{}_{i}, \end{array}$$

where each *x*_*i*_ is a set of patterns and *y*_*i*_ is the label to each example in dataset. A classifier classifies based on the positive and negative examples experienced in the training data, so that given a set of new feature patterns it can predict the corresponding label {+1, − 1}. SVM chooses a subset of the training examples that falls closer to the decision boundary to conclude a hyperplane. This subset is named as support vectors.

Given a dataset, SVM finds the decision hyperplane using a hypothesis function:

(2) $$\begin{array}{c} H\left(\vec{x}{}_{i}\right)=\vec{w}{}^{n}\cdot \vec{x}{}_{i}+b=0, \end{array}$$

where $\vec{w}\in R^{n}$ and *b* ∈ *R*. The hypothesis $H\left({\vec{x}}_{i}\right)$ here depends on a cost minimization principle. A cost estimation function minimizes the cost error between experienced and predicted labels in the dot product space. But the key to SVM is its cost function finding an *optimum hyperplane* by also minimizing the parameter $\vec{w}$ for the hypothesis function

(3) minimizew,b⁡ 12||w||2subjecting yi·((w·xi)+b)⩾1, i=1,…,m.

By minimizing the parameter $\vec{w}$ along with the cost error, it finds a maximum possible distance of the hyperplane to its support vectors. This leads to the optimum hyperplane [20].

SVM learns by applying structural risk minimization principle [21]. As a classifier SVM shows low bias and high variance [16]. One of the highlights of SVM is its ability to apply kernel methods to implicitly map data into the required dot product space.

### 3.2. Kernel Method

The data points fetched to the classifier are in the form of feature patterns. These feature patterns are used to generalize the data points. But defining an explicit feature set to the problem can be huge and tedious, whereas a kernel function helps in implicitly generalizing the data points with a new set of features, that is, in another dimension.

A kernel function maps the given feature space to a higher dimensional space. To a classifier given a new pattern *x* ∈ *y*, the corresponding *y* ∈ {+1, −1} has to be predicted. This needs a similarity measure to be mapped between *x* and *y*. For this a kernel function is required that returns a real number by characterizing the similarity between given data points *x* and *x*′:

(4) $$\begin{array}{c} k:X*X\to R,\\ \left(x,x^{\prime }\right)\to k\left(x,x^{\prime }\right). \end{array}$$

This kind of similarity measure is generally computed using a dot product:

(5) $$\begin{array}{c} \left(x,x^{\prime }\right)=\overset{N}{\underset{i=1}{\sum }}{\left(x\right)}_{i}{\left(x^{\prime }\right)}_{i}. \end{array}$$

For this similarity function using dot product space to work, the data has to be normalized to 1 and has to be mapped to the dot product space. This requires a mapping function *ℏ* : *X* → *F*, where *F* is a kernel space. So from (4) and (5) a kernel function can be represented as

(6) $$\begin{array}{c} k\left(x,x^{\prime }\right)=\left(\hbar \left(x\right)\cdot \hbar \left(x^{\prime }\right)\right). \end{array}$$

### 3.3. Tree Kernel

Tree kernels are intended kernel functions for semantic parse trees. It identifies trees in the form of tree fragments. This way it can identify trees without explicitly considering the entire feature space. Tree fragments are nothing but the different substructures of a tree, where each terminal of the grammar is associated with the tree leaves. In the tree kernel a syntactic parse structure is considered. The popular tree kernel methods used are *subtrees (STs)*, *subSet trees (SSTs)*, and *partial trees (PTs) kernel*.

#### 3.3.1. Subtree

**Figure 1:**
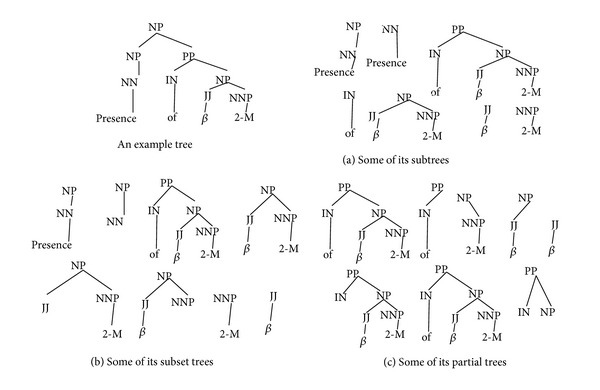
Tree kernels.

A subtree is considered as a constituent sub tree from a node in the original tree. It consists of all of its descendants from the current node in the original tree. Of a tree the total number of subtrees can be generated are much lesser than the substructures generated using SST and PT.  Figure 1(a) shows some of STs of sentence “Presence of beta 2-M.”

#### 3.3.2. Subset Tree

SSTs are more generalized than the ST structures. But it has been constrained that an SST should not break any grammatical rule. That is why the tree substructures of SST may or may not contain the tree leaves but it has to have its entire preterminal nodes in candidate trees.  Figure 1(b) shows eight SSTs (there are more) of the example sentence.

#### 3.3.3. Partial Tree

A partial tree structure does not have the constraint of including all non-terminals like SST. It contains substructures which are partial structures of even SSTs. Figure 1(c) shows eight partial trees of given sentence. The partial trees may or may not contain all the preterminals of constituent subtrees. Of any given sentence the possible PTs are much more than SSTs.

The associated kernel function measures the similarity between two trees by counting the number of their common subparts. The above kernel space is then converted to equivalent vectors of *R* ^*n*^ using the mapping function. And the distance between trees are found by the classifier into the dot product space using the kernel function. By this a kernel function identifies wether tree subparts (common to both trees) belong to the feature space that we intend to generate [7].

In general SST kernel shows better accuracy than ST and PT kernels. The PT shows lower accuracy than the SST but shows a better accuracy on dependency structures [8].

### 3.4. The Proposed Sliding Tree Kernel

Most of the existing NER systems which are built using feature vectors use word syntactic and shallow parse information for classification. An entity name in different contexts can designate different entity classes. Therefore the information of semantic correlations also plays a major role in classification of an entity.

A semantic parse structure using parts-of-speech information is built on a complete sentence. Therefore a tree kernel is subjected to find the distance between two trees, that is, two sentences. But to perform NER task the distance between words has to be found rather than the sentences. When SST, ST, and PT are used in the kernel space for NER task, the kernel space fills with redundant fragments. This is because consecutive words belonging to the same sentence would share similar fragments. That is why the existing tree kernels are not applicable in task like NE identification. Moreover this leads to performance degradation and longer execution time in NER task.

**Figure 2:**
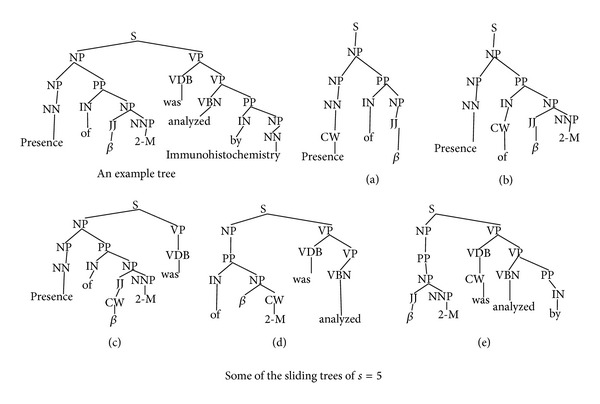
Sliding tree kernel.

To overcome this problem with existing tree kernels a sliding tree kernel (SL) is proposed. This kernel considers a substructure of trees in the form of a sliding window of sliding value “*s*”. Using the sliding value “*s*” a tree is fragmented into the kernel space. These fragments intend to focus a word with its semantic information rather than a complete sentence. Generally an odd sliding value of “*s*” is preferred which semantically centers the focused word. Here the sliding value is required to be chosen initially for a set of experiments. In addition, in the proposed kernel we introduce a word marker in the SL fragments. The marker is placed as the parent node of the current word. In our experiments we have named the marker as “CW” (as current word). So the subset trees which are collected are constrained to have all the preterminals along with the markers. In Figure 2 we have shown the sliding tree fragments for an example sentence: “Presence of beta 2-M was analyzed by immunohistochemistry.” The figure shows five SL trees (out of total eight possible SL fragments: one for each word) forming feature space taking *s* = 5. In return a kernel space is found which intends to generalize the words into the tree kernel space.

Now using the sliding window the constituent subset tree fragments are drawn. These constituent trees are restricted to semantic information of a word through slide window rather than of entire tree. Also, specific marker nodes are introduced in each slide window before few of the selected terminal nodes as pre-terminals.

**Figure 3:**
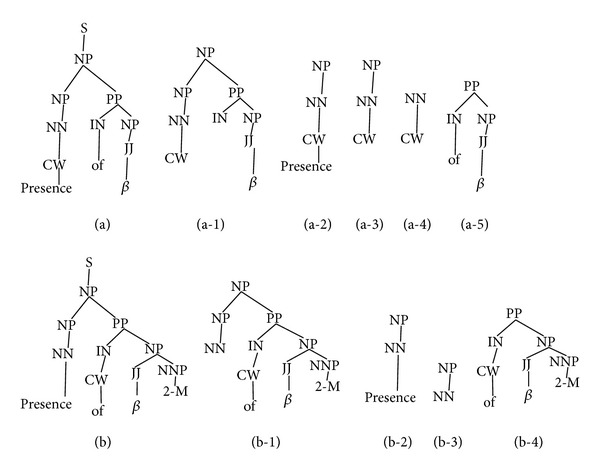
SL constituent trees to word's sliding window.

The SL decreases the structural redundancy of the constituent trees in the kernel space.  Figure 3 shows the constituent trees of sliding windows with markers. The (a) and (b) in Figure 2 show the sliding windows for words “Presence” and “of.” The constituent subset trees generated at the same nodes of original example tree mentioned in Figure 2 show dissimilarity with respect to the words belonging to the same tree. Subtrees (a-1) and (b-1) (in Figure 3) are different even though they start from the same node “NP” which is the left child of node “S” in source example tree (shown in Figure 1). The tree (b-1) also includes word “2-M” as leaf node and its preterminal which (a-1) does not have. And (b-1) has CW marker on the word “of” as preterminal, whereas (a-1) has it on word “Presence.”

This makes SL a task-specific kernel for tasks like NER. It easily identifies the word with generated constituent trees and generalizes for an optimum hyperplane to be found. The distances between the trees are calculated by counting the common substructure of SL trees. As in tree kernel the above kernel space is mapped in the vector spaces of *R* ^*n*^. Then the distance between the words is calculated using the dot product similarity measure. In NER task it shows a competent ability and efficiency both in word identification and boundary detection.

## 4. Experimental Results and Discussion

### 4.1. Training and Test Data

To carry out the experimentations, data from JNLPBA 2004 shared task data is used. The training set contains 20,546 sentences from 2000 abstracts which have about 472,006 tokens, whereas the test set contains 4,260 sentences from 404 abstracts which have about 96,780 tokens. This data for the NER systems to be developed has been preannotated into 5 classes. DNA, RNA, protein, cell-line, and cell-type are the classes. This corpus is extracted from GENIA corpus Version 3.02. For annotation it follows the BIO format, where each class named entity words are annotated into “B-named entity” (for beginning NE word) and “I-named entity” (for rest of NE words). And the words which fall outside these classes are annotated as “*O*” [11].

### 4.2. Evaluation Measure

The evaluation measure has been done by using an *F*-score formula, that is, *F* = (2∗*P*∗*R*)/(*P* + *R*) [11]. This was the standard evaluation measure used in the JNLPBA 2004 shared task, where *P* denotes precision and *R* denotes recall. *P* is the ratio of the named entity correctly identified to the total named entity found in test data. *R* is the ratio of the named entity correctly identified to the actual named entity present in test data. *F* is the harmonic mean between precision and recall.

### 4.3. Performance Using Linear SVM

In linear SVM the linear vector kernel space is explored. Here the base features like word window of *k* value features contribute maximum in performance measures for a NER system. It has been found in the literatures that among word windows *k* value *k* = 5 obtains the maximum *F*-score [13], where as *k* = 7 leads to overfitting and degrades the performance. In Table 1 we show performance score using linear SVM on word features.

### 4.4. Performance of the SL Kernel

SL is a task-specific kernel. So far SL kernel is the only tree kernel which does the complete NER task. Here in Table 2 is shown the effect of the performance in NER task of identifying B-DNA class words with change in “*s*” value in the kernel. The *s* = 7 shows the optimum result among other “*s*” values. With increase in *s* value it leads to overfitting and degrades the system performance.

The tree kernel features the semantic feature and correlation to the words. The baseline system performs NER by using only parts-of-speech information as domain knowledge.  Table 3 projects the complete named entity matched along with the boundary detection performance scores for the classes altogether. It shows *F*-score of 60.60% on the use of sliding tree kernel with *s* = 7.

### 4.5. Comparison with Existing NER Systems Using JNLPBA 2004 Data

In Table 4 we have compared our system with some of the existing systems using JNLPBA 2004 data. Zhou and Su [2] achieved the best accuracy (*F*-score of 72.55) using the data. In that system they have used extensive domain knowledge like name alias resolution, cascaded NE resolution, abbreviation detection and so on. Without the domain knowledge the accuracy of the system is 64.1%. Although our system is not achieving a higher accuracy but without using any domain knowledge it shows a competent performance.

## 5. Conclusion

In this paper we have proposed a NER task-specific kernel function. The proposed kernel function is basically a modification of the existing tree kernel. But it is capable of handling the complete NER task, which the conventional tree kernel is incapable of doing. The proposed kernel is achieving a reasonable accuracy without the use of any domain-specific knowledge. The performance of the system can be improved further by using proper domain-specific information. The task can also be viewed as a two-phase process as done in the literature and the kernel can be applied in both phases separately. These lead the future scopes of work using this kernel.

## Figures and Tables

**Table 1:** Performance of overall NER system on linear SVM using word features.

Feature	Precision%	Recall%	*F*-score%
Word window 5	54.89%	57.34%	56.09%
Word window 7	53%	54.85%	53.91%

**Table 2:** Performance of different *s* values of SL kernel on class B-DNA.

Class	SL tree kernel with changing “*s*” value	Precision%	Recall%	*F*-score%
B-DNA	*s* = 5	79.61%	42.89%	55.75%
B-DNA	*s* = 7	82.44%	42.7%	56.26%
B-DNA	*s* = 9	82.17%	41.47%	55.12%

**Table 3:** The overall NER system performance using proposed kernel of *s* = 7.

SL tree kernel *s* value	Precision%	Recall%	*F*-score%
*s* = 7	72.63%	51.99%	60.60%

**Table 4:** Our system compared with existing systems.

System	ML approach	Domain knowledge	*F*-score%
Normal SVM	Linear SVM	—	56.09%
Our system	SVM with SL	—	60.60%
Zhou and Su (2004) [2] final	HMM, SVM	Resolution of name alias, cascaded NEs, and Abbreviations; dictionary; POS	72.55%
Zhou and Su (2004) [2]	HMM, SVM	(baseline)	64.1%
Song et al. (2004) [15] final	SVM, CRF	POS information, phrase, and virtual sample	66.28%
Song et al. (2004) [15]	SVM	(baseline)	63.85%
Saha et al. (2010) [18] final	Composite kernel	—	67.89%
